# A rare complication of acute lower limb ischemia post coronavirus disease 2019 infection in a healthy pediatric patient: case report

**DOI:** 10.1186/s12887-023-04454-8

**Published:** 2023-12-09

**Authors:** Arwa Badr, Khayria AlSofyani, Yara AlGoraini

**Affiliations:** 1 Department of Pediatric Rheumatology, Maternity and Children Hospital, Makkah, Saudi Arabia; 2 Peditric Emergency Medicine Department, King Fahad Medical City, Riyadh, Saudi Arabia

**Keywords:** Lower limb ischemia, Post-COVID-19, Pediatric, Case report

## Abstract

**Background:**

Coronavirus Disease 2019 (COVID-19) is a novel respiratory disease that first emerged in 2019. Patients infected with this disease present with a myriad of symptoms. Limb ischemia and hypercoagulability are complications identified in adults. COVID-19-related vasculitis is a known but seldom reported complication in pediatric patients, and the treatment approach is still not well established.

**Case presentation:**

We report the case of a healthy four-year-old female with a history of COVID-19 who developed acute lower limb ischemia. This was initially treated as a case of acute snake envenomation by administering snake antivenom with no improvement. She eventually developed lower limb acrocyanosis with an inability to ambulate. The patient was started on interleukin-6 receptor inhibitors (tocilizumab), anticoagulants, and pulse steroid therapy. The patient had complete resolution with the loss of only one toe.

**Conclusion:**

Identification of thromboembolic complications in pediatric patients with no comorbidities and a history of COVID-19 can be difficult. Early recognition and treatment have a major impact on morbidity and can increase the likelihood of limb salvage.

## Background

Coronavirus Disease 2019 (COVID-19) is caused by Severe Acute Respiratory Syndrome Coronavirus 2 (SARS-CoV-2), which was discovered in December 2019 and was declared a pandemic by the World Health Organization (WHO) on March 2020 [1]. Since December 2019, when the first case of coronavirus disease was reported, there has been a constant evolution in the clinical presentation of the disease from simple viral-like illnesses such as fever, sore throat, and myalgia to Acute Respiratory Distress Syndrome (ARDS), multiple organ failure, and microthrombosis [2].

Elevated coagulation factors, combined with cytokine overproduction, increase the risk of microthrombosis, vascular hyperpermeability, Disseminated Intravascular Coagulation (DIC), and multiple organ failure.

COVID-19-Associated Coagulopathy (CAC) includes arterial and venous thromboembolism (VTE). Arterial thrombosis in patients with COVID-19 can manifest in various ways, ranging from blue toe syndrome to limb-threatening acute limb ischemia (ALI) [3–6].

ALI is defined as a sudden decrease in arterial perfusion that threatens limb viability and occurs within 14 days of symptom onset [3]. Because there is insufficient time for neovascularization to compensate for the loss of perfusion, ALI threatens limb viability within a short period. Sudden ischemia affects the skin, muscles, and nerves of the limbs, resulting in necrosis and tissue death. Immediate revascularization is required to preserve limb viability [3]. The annual incidence of ALI is about 1.5 cases per 10,000 people [7]. COVID-19 can cause arterial thrombosis owing to endothelial injury, and ALI was five times more common in COVID-positive patients than in COVID-negative patients [7].

Here, we report a rare case of a previously healthy pediatric patient who developed bilateral lower ALI post-COVID-19 infection. Due to the varied clinical presentations and paucity of pediatric literature, pediatricians should be aware of this complication.

## Case presentation

**Fig. 1:**
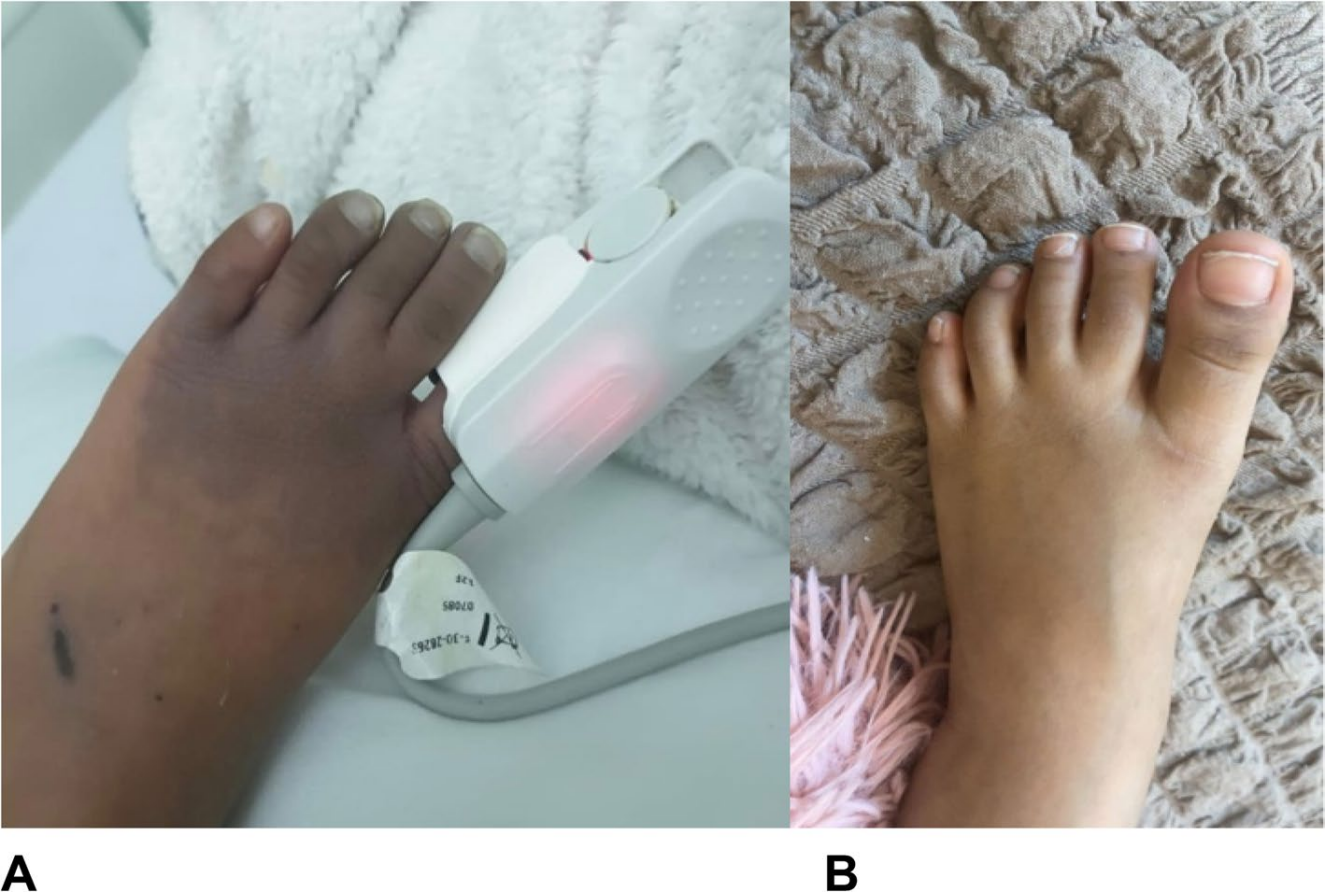
**A** Dorsum of the left foot with cyanotic discoloration on the left toes developed gradually over one week. **B** Complete resolution of the cyanotic discoloration of the left foot a couple of days later

Our patient was a 4-year-old previously healthy female who came in at a peripheral hospital for intense pain in the left foot and one-week history of cyanotic discoloration in her left toes. The discoloration progressed rapidly and involved the dorsal surface of the left foot (Fig. 1A). The working impression at the peripheral hospital at first was a possible snakebite since the family lived in a rural area were the incidence of scorpions or snakes was highly considered, also the history had poor reliability. They introduced five vials of snake anti-venom along with acetaminophen for pain and kept her for four hours for observation and eventually discharged her with instructions on when to return to the Emergency Department (ED) for further follow-up. The left foot discoloration has been completely resolved in two days after first presentation to the ED. However, within five days after the resolution of the left foot discoloration, the right foot had exhibited the discoloration at the toes where the patient presented to the ED.

**Fig. 2:**
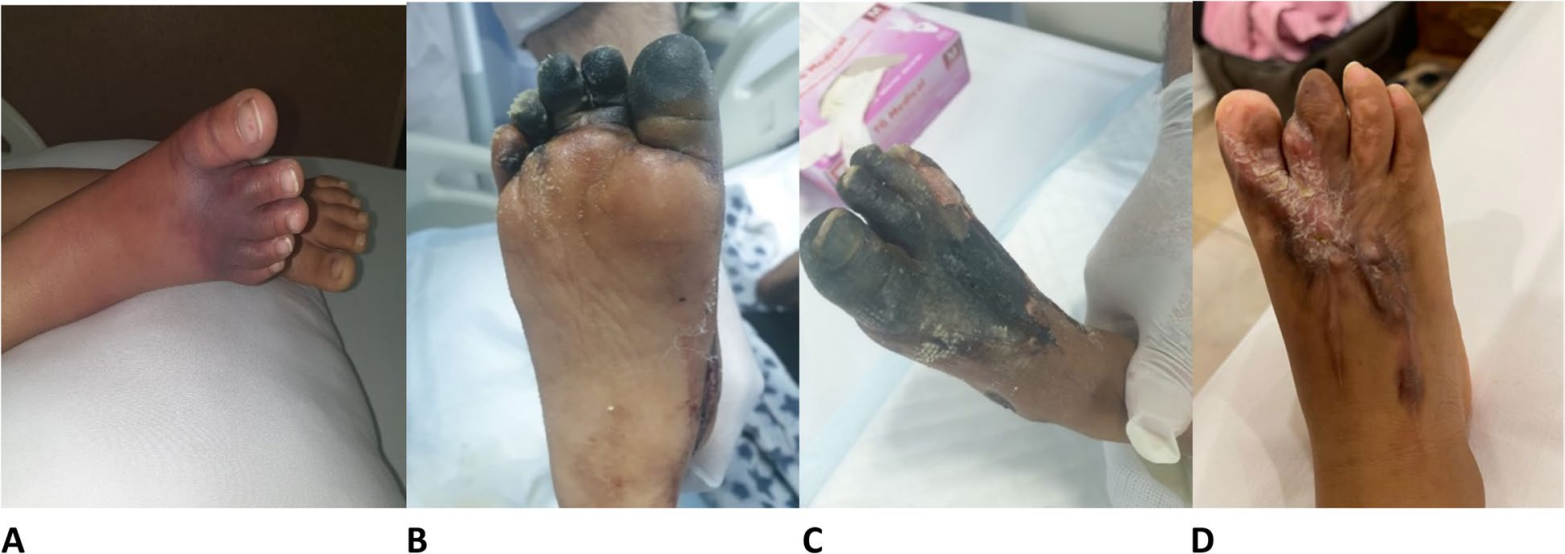
**A** Right foot at the first week of presentation at our emergency department. **B** and** C**: Right foot after ten days of the initial symptoms and after admission to our hospital. **D**: After a four-month recovery period, the patient lost a second toe due to autoamputation

Her left foot and toes had completely resolved within a couple of days (Fig. 1B) but her right foot started to develop the same symptoms, bringing her back to the ED (Fig. 2A).

The patient had no history of fever, weight loss, or appetite changes. There was no history of rashes, and the other limbs or joints were unaffected. She had no history of headaches, blurry vision, chest pain, palpitations, and respiratory symptoms. She had an unremarkable antenatal and neonatal history. Past medical and family histories were negative for rheumatological diseases or similar episodes. She was not on any medication nor had she received any vaccinations recently. Both parents were college graduates with good socioeconomic status.

There was a history of COVID-19 infection one year prior, where she developed a mild upper respiratory tract infection that did not require hospitalization and was managed conservatively. Two days before her presentation to our ED, her father had a positive nasopharyngeal swab test for COVID-19 reverse transcription polymerase chain reaction (RT-PCR) as he had an upper respiratory tract infection for the past week.

Upon examination, the child was alert, active, and anxious but was not in distress. She noted severe pain in her right foot and an inability to ambulate. Her vital signs were as follows: temperature, 37.2 °C; heart rate, 110 bpm; blood pressure, 105/65 mmHg; respiratory rate, 23 bpm; and glucose, 4.5 mmol. Her oxygen saturation level was 100% in ambient air. Lower limb examination showed five cyanotic toes on the right foot that extended to the dorsal and plantar surfaces (Fig. 2A). The pulses of the dorsalis peds, posterior tibia, and popliteal arteries were palpable. The oxygen saturation of the toes was normal. The results of cardiorespiratory and neurological examinations were unremarkable.

Initial laboratory evaluation showed mild thrombocytosis, high alanine transaminase (ALT) level, mildly high direct bilirubin level, and a significantly high erythrocyte sedimentation rate (ESR) (Table 1).

**Table 1:** Patients’ laboratory results

Laboratory		Result	Normal range	Comment
Hematology	White blood cells	8.64 × 10^3^/µL (5–10 × 10^3^/µL)	5–10 × 10^3^/µL	Normal
	Neutrophils	55% (40–80%)	40–80%	Normal
	Monocyte	10.3% (2–10%)	2–10%	High
	Hemoglobin	12.2 g/dL (11–15.5 g/dL)	11–15.5 g/dL	Normal
	Hematocrit	85.400 Fl (76–87 Fl)	76–87 Fl	Normal
	Platelet	610 × 10^3^/µL (170–490 × 10^3^/µL)	170–490 × 10^3^/µL	High
	Protein S	1.2 U/ml	0.60–1.60 U/ml	Normal
	Protein C	0.6 U/ml	0.72–1.23 U/ml	Low
Coagulation	Prothrombin Time	11.3/sec (11–16/sec)	11–16/sec	Normal
	Partial thromboplastin time	1.0 (0.8–1.3)	0.8–1.3	Normal
	International Normalized Ratio	27.3/sec (23–37/sec)	23–37/sec	Normal
Liver function test	Alanine Transaminase	80 IU (0–55 IU)	0–55 IU	High
	Aspartate Aminotransferase	33 IU (5–34 IU)	5–34 IU	Normal
	Albumin	42 g/L (38–54 g/L)	38–54 g/L	Normal
	Alkaline phosphatase	125 IU (156–369 IU)	156–369 IU	Low
	Direct bilirubin	6 µmol/L (0–5 µmol/L)	0–5 µmol/L	High
	Total bilirubin	11.6 µmol/L (3–22 µmol/L)	3–22 µmol/L	Normal
Renal function test	Creatinine	45 µmol/L (27–62 µmol/L)	27–62 µmol/L	Normal
	Uric acid	218 µmol/L (120–320 µmol/L)	120–320 µmol/L	Normal
	Urea	3.0 mmol/L (1.8–6.4 mmol/L)	1.8–6.4 mmol/L	Normal
	Sodium	136 mmol/L (138–145 mmol/L)	138–145 mmol/L	Low
	Potassium	4.7 mmol/L (3.4–4.7 mmol/L)	3.4–4.7 mmol/L	Normal
COVID serology	Immunoglobulin G (IGG) and Immunoglobulin G (IgM)	Positive		
Inflammatory Markers	Erythrocyte Sedimentation Rate	62 mm/h (1–13 mm/h)	1–13 mm/h	High
	C-reactive protein	0.1 (< 0.5)	< 0.5	Normal
	Fibrinogen	4.51 g/L (1.57–4 g/L)	1.57–4 g/L	Normal
	D-dimer	2.31 mg/L	< 0.5 mg/dl	High
	Ferritin	141.2 ng/mL (10–200 ng/mL)	Females, 10–200 ng/mL	Normal
	Complement 3 (C3)	90 mg/dl	80–178 mg/dl	Normal
	Complement 4 (C4)	24 mg/dl	12–42 mg/dl	Normal
	C -Antineutrophil Cytoplasmic Antibodies (C-ANCA) and peripheral antineutrophil cytoplasmic antibodies (P-ANCA)	Negative		
	Antistreptolysin O (ASO) titer	Negative		

**Fig. 3:**
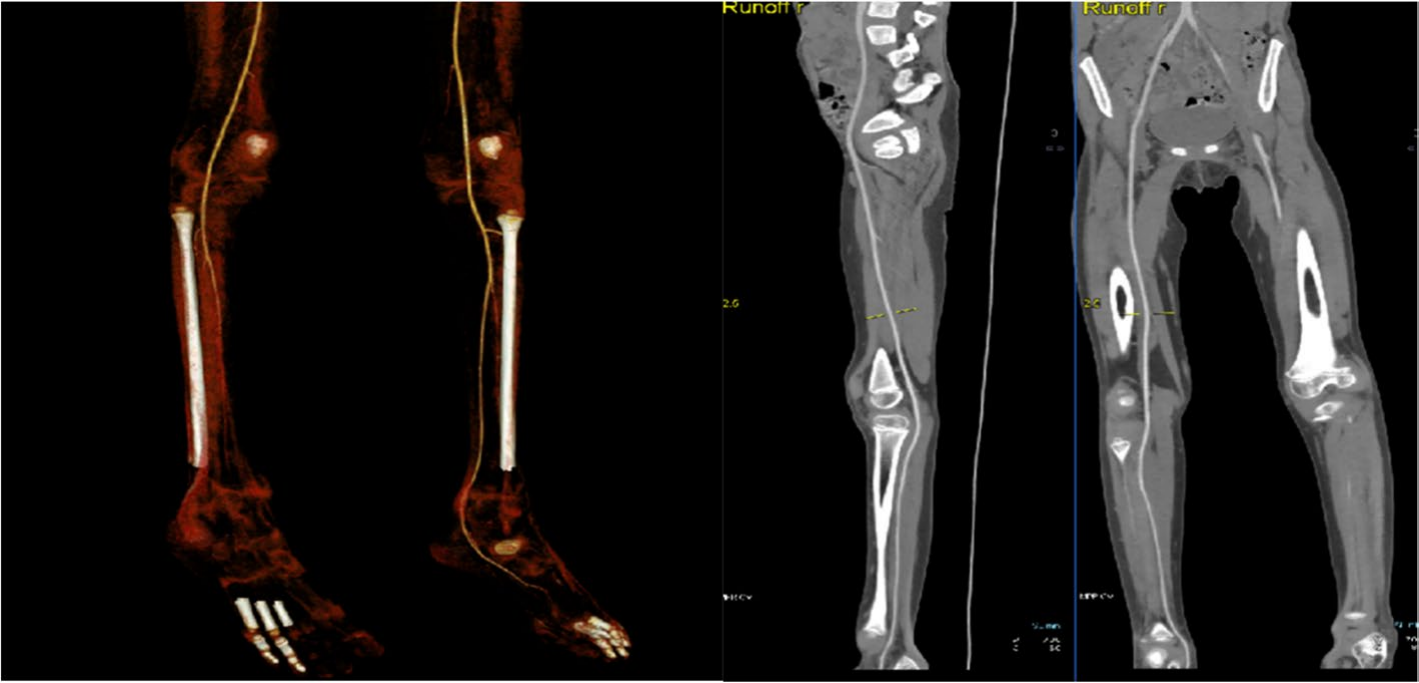
Computed tomography angiogram of the lower limbs showed a mild to moderate attenuation of the right anterior and posterior tibial and dorsalis pedis

The patient was then admitted for further investigation. Advanced imaging was performed using a computed tomography angiogram of the lower limbs, which showed mild-to-moderate attenuation of the right anterior and posterior tibial and dorsalis pedis arteries (Fig. 3).

COVID-19 was diagnosed using Reverse transcription polymerase chain reaction (RT-PCR) testing and serology was positive for Immunoglobulin G (IGG) and Immunoglobulin G (IgM). The patient was placed on airborne precaution and started on pulse steroids protocol 30 mg per Kg infusion over 3 h given as an in-patient which followed by oral prednisolone at dose of 1 mg per kg once daily for five days as an outpatient then it was discontinued. In addition to two doses of IL-6 (12 mg/kg; tocilizumab). Also, received single dose of intravenous immunoglobulin (2 g per kilogram). Multiple specialties were consulted during admission. The hematology service recommended to measure serum protein S and C which were found to be not significantly elevated above the normal limit. Also, they recommend that the patient start Enoxaparin at a dose of 0.5 mg per kg per dose every 12 h subcutaneously for twenty-one days as recommended by the in-house consultant hematologist and was discontinued before discharge. Pediatric orthopedics advised fasciotomy to preserve homeostasis. In addition, vascular surgeons were consulted and advised that no further interventions were needed, as there was only a mild attenuation. On the tenth day of her initial symptoms, the patient's right foot became darker and charcoal in color with preserved pulses and oxygen saturation on the toes (Fig. 2B and C).

On the fourteenth day of admission, after medical and surgical interventions, her right foot and toes markedly improved in color, the pain subsided, and she was able to ambulate. The patient was discharged during the follow-up. The patient was seen one month later with significant improvement on the dorsal surface of the foot and some fingers. Four months later, the patient lost her second toe due to autoamputation as a complication (Fig. 2D). However, she was doing well with complete resolution of the signs and symptoms of her right foot and was able to walk without pain.

## Discussion

The COVID-19 pandemic is constantly evolving. Fever, myalgia, cough, and dyspnea are common clinical manifestations of SARS-CoV-2 infection, along with headache, diarrhea, nausea, and vomiting [3]. The state of hypercoagulability caused by SARS-CoV-2 has been shown to manifest in a wide range of presentations ranging from asymptomatic infection to critical disease. Although respiratory symptoms predominate in patients with COVID-19, thrombosis can also occur. There have been reports of ALI observed mostly among adults with comorbidities [2–5, 7] and only one case has been reported in an adolescent [6], but to our knowledge, no cases at the time of writing of this case has been reported in younger age groups. This is the first reported case of a four-year-old with ALI due to COVID-19.

ALI is defined as a sudden decrease in arterial perfusion of a limb that threatens its viability. If the symptom duration is less than two weeks, the clinical presentation is considered acute [8]. Pain, pallor, paralysis, decreased pulse rate, paresthesia, and poikilothermia are the classic clinical features of patients with ALI. Symptoms range from new or worsening intermittent claudication to severe pain at rest, paresthesia, muscle weakness, paralysis, and even gangrene, and may become apparent within minutes, hours, or days [9]. We discovered acute ALI symptoms in this patient, including cyanosis and paralysis. Based on Rutherford classification, the patient was diagnosed with Rutherford stage IIb disease.

Similar case was reported in Pakistan, Lahore, where an elderly patient with comorbidities presented with fever and lower leg discoloration in which became a non-salvageable limb and required subsequent amputation of the affected limb [2].

Several recently published articles have reported the occurrence of arterial thrombotic events in COVID-19-positive patients with no history of peripheral arterial disease [2, 3].

The mechanism underlying ALI in patients with COVID-19 is complex. It is attributed to the angiotensin-converting enzyme 2 receptor, which is found in almost all tissues of the body, that the virus can use to enter the host cells leading to the release of damage-associated molecular pattern, which results in the release of the inflammatory cascade and proinflammatory cytokines, eventually triggering a thromboinflammatory process [10].

The therapeutic strategy is based on the presence of a neurological deficit, location, Rutherford class, duration of ischemia, comorbidities, and risks and outcomes associated with the therapy. Patients with clinically suspected ALI should be admitted to the ED for immediate diagnosis and treatment. Anticoagulation therapy with Enoxaparin is administered promptly to prevent thrombus propagation and preserve microcirculation. Enoxaparin inhibits the development of cytokine storm and has competitive binding activity to the coronavirus, significantly reducing pathogen activity by inhibiting cell penetration [3]. Depending on the Rutherford classification, revascularization was required. Our patient had class II ALI, in which the limb was saved with immediate intervention using fasciotomy and Enoxaparin. However, in a case reported in Indonesia, the Rutherford classification was IIb, and the patient was treated with anticoagulant therapy with unfractionated Enoxaparin and referred to other hospital to perform a thrombectomy [3].

However, in a case report in Greece, the authors attempted to combine thrombolytic therapy with recombinant tissue plasminogen activator (rt-PA) and immunosuppressive therapy with IL-6 (tocilizumab) in a patient who was admitted with acute respiratory failure secondary to COVID-19 pneumonia, who later developed ALI in his lower limb digits. However, after starting the management patient showed poor prognosis and had major amputations in which he died due to respiratory failure [11]. The patient, wherein we used a combination of Enoxaparin, tocilizumab, and pulse steroid therapy, later showed complete resolution of symptoms in all previously involved digits [12]. To our knowledge, this is the first report on the use of a combination of thrombolytic and anti-inflammatory therapy for treating COVID-19-induced ALI in pediatric patients reported in the international literature.

The variety of COVID-19 symptoms has increased rapidly since 2020. Initially, attention was focused on respiratory problems; however, other symptoms have recently surfaced, including COVID-19-induced myocarditis, arthritis, liver damage, and encephalitis. ALI is challenging to treat because the limb cannot be saved beyond a certain period. Since everyone focuses on the patient's respiratory condition, especially pediatric patients, such complications may be overlooked. By sharing this, we hope to share our experience and contribute to the research on how a case of COVID-19-induced ALI in pediatric patients was handled in a developed country.

## Conclusion

Identification of thromboembolic complications in pediatric patients with no comorbidities and a history of COVID-19 can be difficult. Early recognition and treatment have a major impact on morbidity and maximize the likelihood of limb salvage. We suggest that patients with COVID-19-induced ALI should receive a combination of anticoagulation, immunotherapy, and pulse steroid therapy. In our case, combination therapy may have improved the outcome for the right foot. However*,* further research is needed.

## Data Availability

Data that support the findings in the current study are available from the corresponding author on reasonable request.

## Abbreviations

COVID-19: Coronavirus Disease of 2019
ALI: Acute Lower Limb Ischemia
IL-6: Interleukin-6
SARS-CoV-2: Severe Acute Respiratory Syndrome Coronavirus 2
WHO: World Health Organization
ARDS: Acute Respiratory Distress Syndrome
DIC: Disseminated Intravascular Coagulation
CAC: COVID-19 Associated Coagulopathy
VTE: Venous Thromboembolism
ED: Emergency Department
RT-PCR: Reverse Transcription Polymerase Chain Reaction
ALT: Alanine Transaminase
ESR: Erythrocyte Sedimentation Rate
ACE: Angiotensin-Converting Enzyme 2
rt-PA: Recombinant Tissue Plasminogen Activator
